# Epidemiology and clinical presentation of glaucoma in a referral facility in Ghana: Any lessons for public health intervention?

**DOI:** 10.1371/journal.pone.0245486

**Published:** 2021-01-15

**Authors:** Samuel Kyei, Patience Asantewaa Obeng, Michael Agyemang Kwarteng, Frank Assiamah

**Affiliations:** 1 Department of Optometry and Vision Science, School of Allied Health Sciences, College of Health and Allied Sciences, University of Cape Coast, Cape Coast, Ghana; 2 Department of Optometry, Faculty of Science and Engineering, Bindura University of Science Education, Bindura, Zimbabwe; The University of Melbourne, AUSTRALIA

## Abstract

The purpose of this study was to evaluate the epidemiological and clinical profile of patients living with glaucoma and receiving care in a tertiary eye center in Ghana. This was a hospital-based retrospective study of clinical records of glaucoma patients from January 2010 to December 2019. The study involved collating demographic information of patients, clinical presentation, and the management of glaucoma. A total of nineteen thousand (19,000) charts were retrieved from the eye center’s archives. Out of these, 660 (3.5%) records of patients qualified for the study and were analyzed. There were 398 (60.3%) males and 262 (39.7%) females. Their ages ranged from 9 to 86 years (mean age = 47.30; SD ± 16.86 years). The averages of ocular parameters of 1,320 eyes (660 patients) were visual acuity = 0.26 ± 0.55 logMAR; intraocular pressure: 17.31 ± 6.11 mmHg; cup-to-disc ratios: 0.67 ± 0.17 D; and the average retinal nerve fibers thickness was 95.03 ± 21.74 *μ*m. The mainstay of treatment was the sole use of medication. Males were the major group receiving glaucoma care at the tertiary level. Glaucoma cases included juveniles but the mean age suggests most were of adult-onset. Socio-demographic characteristics affected the diagnosis and management of glaucoma among patients receiving care at a referral center. Public health, stakeholders, and policymakers’ interventions can help identify individuals with glaucoma.

## Introduction

Glaucoma is the leading cause of irreversible blindness but ranked second to cataract in the global causes of blindness [1, 2]. Glaucoma is an optic neuropathy associated with characteristic structural damage to the optic nerve and visual dysfunction that may be caused by various pathological processes and in which intraocular pressure, (IOP) is a key modifiable factor [3]. It is usually classified based on etiology, the anatomy of anterior chamber angle, time of onset, and pathogenesis [4, 5].

Primary open-angle glaucoma (POAG) is the commonest type of glaucoma among people of African descent [6–8]. The incidence of POAG is associated with age [6, 9] and people of African descent are at a higher risk of POAG than non-Africans [10]. The economic cost associated with a visual impairment from glaucoma is considerable as it affects the productivity of the individual and the nation as a whole. Glaucoma management remains a challenge to eye health services as the majority of the population are unaware of their status.

In practice, comprehensive glaucoma care in Ghana happens at the tertiary referral eye care facilities, as most detected cases from primary and secondary care facilities are referred for expert care. The prevalence of glaucoma cases reporting to a referral facility can therefore serve as a fair benchmark for evaluating the eye care-seeking behavior and the coverage of care for glaucoma. Available studies [11–18] in Ghana have amply dealt with the prevalence, risk factors, and to some extent the genetics of glaucoma but there remains a paucity of information on the epidemiology and clinical profile of patients living with glaucoma and receiving care in health care facilities. There is a recent study [19] on the epidemiological data among patients with Glaucoma and receiving care in a referral facility in the Ashanti region. This 5-year retrospective study involving 311 out of 1100 records suggests low patronage of the facility or missing records which tends to affect the data presented [19].

Moreover, knowing the epidemiological and clinical profile of glaucoma patients cannot be underestimated since it helps in health planning, monitoring, and resource allocation. It is against this background that this study aimed to determine the epidemiological characteristics and clinical presentation of glaucoma among patients visiting a referral facility in Ghana to draw lessons for public health interventions.

## Materials and methods

### Study setting

This study was carried out at the premises of the Bishop Ackon Memorial Christian Eye Center, Cape Coast. The center is the most utilized Christian health eye facility in the Cape Coast metropolis of Ghana.

### Study design

This was a clinic-based retrospective study of patients diagnosed with glaucoma and receiving care at the Christian Eye Center from January 2010- December 2019.

### Sampling technique

The sampling method was non-probability convenience sampling. The sampling method was based on the fact that the study involved all patients with glaucoma visiting the center during the study period.

### Inclusion and exclusion criteria

The study included all patients with records of diagnosed glaucoma and receiving care at the Christian Eye Center including those who had undergone laser surgery, those on anti-glaucoma medications, or those who have undergone filtration surgery. The diagnosis of glaucoma was based on the presence of a glaucomatous optic nerve head changes i.e. diffuse or localized rim thinning and disc hemorrhage, bayoneting, notch, baring, or vertical cup-to-disc ratio of 0.5 or difference in cup disc ratio of more than 0.2 in the two eyes, in the absence of significant difference in disc size, and visual field defects that matched with the RNFL defects, optic nerve head abnormalities and gonioscopically open or close angles. On the other hand, patients with ocular hypertension but showing no changes in optic nerve head or visual function abnormalities were excluded from the study. All patient records with SITA standard 24–2 perimetry (Carl Zeiss Meditec Inc., Dublin, CA, USA) within the defined reliable visual field test of fewer than 20% of fixation losses, false positive or false negatives were included. The OCT results of the RNFL were obtained using the RTVue system Version #A6, 8,0, 27 (Optovue, Inc., Fremont, CA, USA) with signal strength intensity of at least 50%. Patients’ records with a history of ocular comorbidities such as macular degeneration, retinitis pigmentosa, hypertensive retinopathy, diabetic retinopathy, refractive error of ± 4 dioptres (D) sphere and/or astigmatism of 3D, and significant cataract that affect vision were excluded as they could affect the validity of the ocular imaging reports.

### Ethical consideration

The study adhered to the tenets of the Declaration of Helsinki and was approved by the Institutional Review Board of the University of Cape Coast (UCCIRB/CHAS/2019/187). Permission to access the facility and patient records was obtained from the management.

### Data collection procedure

Data collection involved the use of a data extraction sheet to extract information on socio-demographics, and clinical profile of patients. The data on socio-demographics of patients included sex, age, ethnicity, religion, and occupation. The clinical profile recorded included presenting visual acuity, IOP, cup-to-disc ratios (CDR), cup volume, cup-to-disc area, vertical cup-to-disc, rim area, disc area, glaucoma hemifield test, visual field indices, and average retinal nerve fiber layer (RNFL) parameter, and management modality to glaucoma.

### Statistical analysis

Data were analyzed using the IBM SPSS version 21 (SPSS Inc., Chicago, USA). Categorical data were presented as frequencies. Descriptive statistics were computed for all variables after the data have been screened and the normality test carried out.

## Results and discussion

Nineteen thousand (19000) charts were retrieved from the eye center’s archives of which 660 were patients with glaucoma. The glaucoma patients ages ranged from 9 to 86 years (mean age = 47.30; SD ± 16.86 years). All the 660 participants presented with bilateral cases of glaucoma (1320 eyes), 398 (60.3%) were males and 262 (39.7%) were females. Among the patients, 310 (47%) resided in an urban area, 50.9% were Akans, 89.5% were Christians followed by Islam (9.9%) (Table 1).

**Table 1:** Distribution of socio-demographics according to sex.

Variables		Sex of patients		Total (%)
		Male	Female	
**Residence**	Rural	147	97	**244** (37.0)
	Peri-urban	60	46	**206** (16.1)
	Urban	191	119	**310**(47.0)
**Ethnicity**	Akan	201	135	**336 (50.9)**
	Guan	81	51	**131(19.8)**
	Ewe	57	40	**97(14.7)**
	Ga-Adangbe	59	37	**96** (14.5)
**Religion**	Christianity	352	239	**591** (89.5)
	Islam	42	23	**65** (9.9)
	Traditional	2	-	**2** (0.3)
	Atheist	-	2	**2** (0.3)
**Occupation**	Self-employed	147	86	**233** (35.3)
	Civil servant	123	85	**208** (31.5)
	Retired	63	39	**102** (15.5)
	Students	35	27	**62** (9.4)
	Unemployed	14	21	**35** (5.3)
	Farming	13	4	**17** (2.6)
	Military	-	3	**3** (0.5)
**Marital status**	Married	303	189	**492** (74.5)
	Single	78	61	**139** (21.1)
	Divorced	7	9	**16** (2.4)
	Widowed	10	3	**13** (2.0)

### Prevalence of glaucoma

The prevalence of glaucoma at the tertiary eye center during the ten-years was 660 out of 19000 representing 3.5% (95% CI; 3.2–3.7) of the total cases reported to the eye care facility. The common type of glaucoma was the primary open-angle (Table 2). There were no ethnic peculiarities for CDR (P>0.05).

**Table 2:** Distribution of glaucoma according to sex.

Type of glaucoma	Sex		Total (OD, OS)
	Female (OD, OS)	Male (RE, LE)	
** Primary open-angle**	180,015	342,343	517,558
** Normal-tension**	45, 47	56, 55	101, 102
**Total**	262	398	660

OD: oculus dexter, OS: oculus sinister

**Fig 1:**
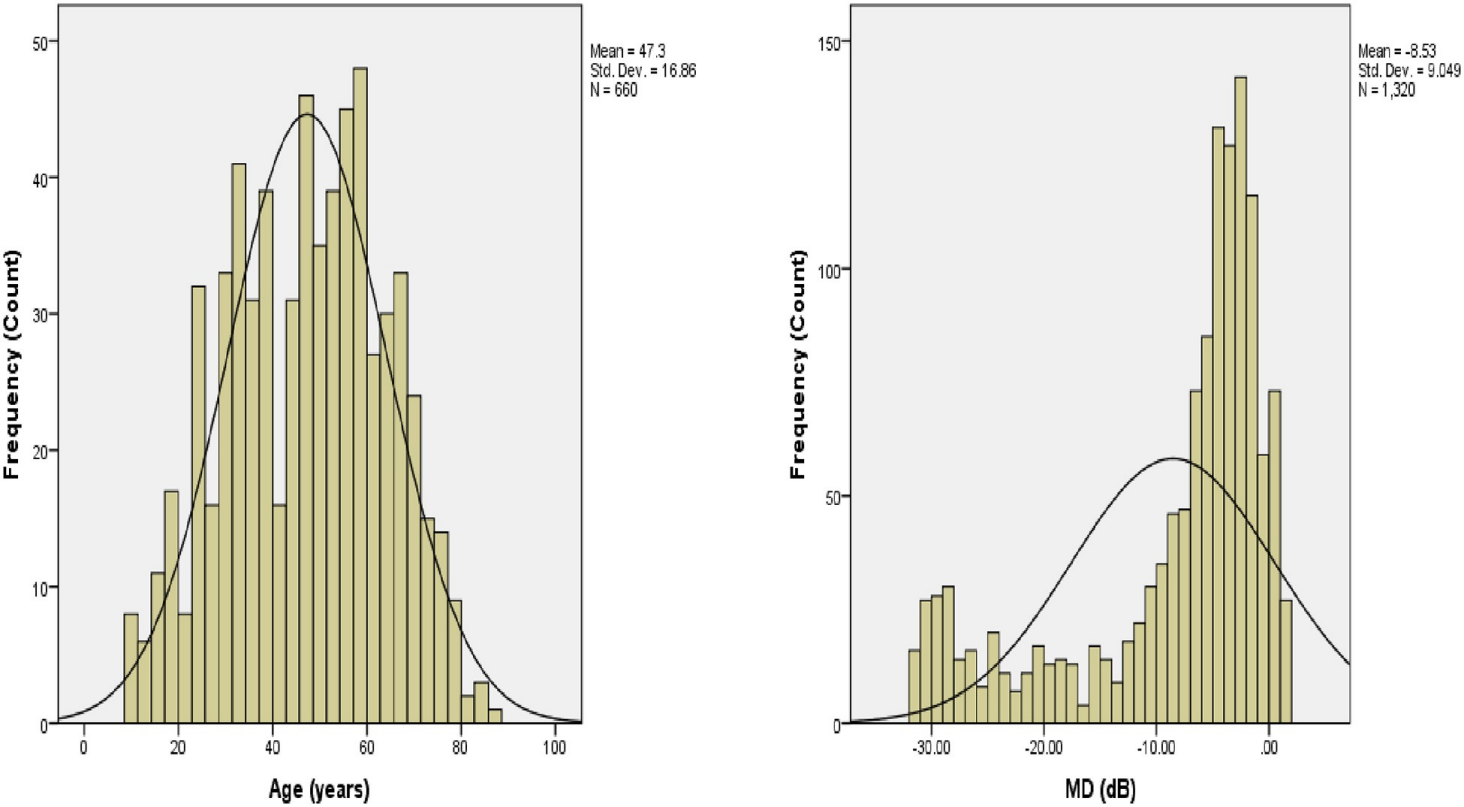
A histogram of the distributions of baseline age (A), baseline MD (B), for a cohort of 1,320 patients from Ghana.

The clinical profiles such as IOP, cup-to-disc ratios, retinal nerve fiber layers among others were computed (Table 3, Fig 1).

**Table 3:** Presentation of average clinical indices of glaucoma.

Clinical index	Sex		Levene’s Test for Equality of Variances	
	Female (n = 262)	Male (n = 398)	F	Sig.
**VA OD (logMAR)**	0.23 ± 0.52	0.30 ± 0.61	2.78	0.096
**VA OS (logMAR)**	0.24 ± 0.55	0.28 ± 0.60	0.71	0.399
**IOP OD (mmHg)**	16.56 ± 5.17	17.80 ± 6.26	11.51	0.001*
**IOP OS (mmHg)**	16.62 ± 5.40	18.12 ± 7.00	11.49	0.001*
**Cup-Disc Ratio OD**	0.64 ± 0.16	0.69 ± 0.18	3.20	0.074
**Cup-Disc Ratio OS**	0.64 ± 0.16	0.70 ± 0.16	0.02	0.877
**Average RNFL (*μ*m) OD**	101.21 ± 18.27	91.80 ± 23.4	24.98	0.000*
**Average RNFL (*μ*m) OS**	99.26 ± 19.67	91.50 ± 22.17	18.00	0.000*
**Superior RNFL (*μ*m) OD**	101.09 ± 20.78	91.14 ± 22.62	12.28	0.000*
**Superior RNFL (*μ*m) OS**	99.50 ± 18.40	92.38 ± 22.40	20.40	0.000*
**Inferior RNFL (*μ*m) OD**	101.63 ± 19.05	92.35 ± 25.04	24.40	0.000*
**Inferior RNFL (*μ*m) OS**	98.83 ± 21.17	90.86 ± 23.43	11.70	0.001*
**Cup Volume OD**	0.47 ± 0.35	0.53 ± 0.40	3.76	0.053
**Cup Volume OS**	0.50 ± 0.38	0.59 ± 0.42	0.88	0.348
**Cup-Disc Area Ratio OD**	0.55 ± 0.30	0.64 ± 0.52	3.09	0.079
**Cup-Disc Area Ratio OS**	0.56 ± 0.24	0.60 ± 0.20	0.00	0.989
**Vertical Cup-Disc Ratio OD**	0.69 ± 0.16	0.75 ± 0.17	2.07	0.151
**Vertical Cup-Disc Ratio OS**	0.71 ± 1.6	0.75 ± 0.18	0.49	0.484
**Rim Area OD**	1.19 ± 0.48	1.01 ± 0.50	0.18	0.672
**Rim Area OS**	1.22 ± 0.51	1.05 ± 0.54	2.38	0.124
**Disc Area OD**	2.50 ± 0.63	2.40 ± 0.69	0.41	0.521
**Disc Area OS**	2.50 ± 0.70	2.43 ± 0.64	2.26	0.133
**Mean deviation (dB) OD**	-6.65 ± 7.46	-9.55 ± 9.55	31.98	0.000*
**Mean deviation (dB) OS**	-6.92 ± 7.99	-9.79 ± 9.78	26.75	0.000*
**Pattern Std D—OD (dB) OD**	3.47 ± 2.62	4.31 ± 3.35	8.08	0.005*
**Pattern Std D—OD (dB) OS**	3.49 ± 3.21	4.21 ± 2.93	3.28	0.071

OD: oculus dexter, OS: oculus sinister, VA: Visual acuity, IOP: Intraocular pressure, RNFL: Retinal nerve fiber layer, Std D: Standard deviation.

*There was a significant difference between the variables among gender.

The glaucoma hemifield test results were computed in Tables 4 and 5.

**Table 4:** Glaucoma hemifield test according to sex.

Glaucoma Hemifield Test	Sex of Patient		Total (OD, OS)
	Male (OD, OS)	Female (OD, OS)	
**Outside Normal Limit**	186, 188	78, 76	264, 264
**Within Normal Limit**	144, 138	118, 113	262, 251
**Borderline**	38, 43	26, 31	64, 74
**General Reduction in Sensitivity**	30, 29	40, 42	70, 71
**Total**	398	262	660

OD: oculus dexter, OS: oculus sinister

**Table 5:** Distribution of treatment according to sex.

Intervention	Sex		Total
	Female	Male	
**Beta-blocker**	88	92	180
**Alpha-2 agonists**	73	133	206
**Carbonic anhydrase inhibitors**	6	14	20
**Prostaglandin-analogue**	77	127	204
**Surgical (Trabeculectomy)+prostaglandin-analogue**	8	12	20
**Surgical (Trabeculectomy)+Beta-blocker**	10	20	30
**Total**	262	398	660

The mainstay of treatment was through medication only which accounted for 92.4% of the study population as shown in Table 5.

This ten-year clinic-based prevalence study indicated that fewer persons with glaucoma are receiving care relative to the reported prevalence in the population of Ghana [16]. This points to the critical need for a thorough glaucoma screening campaign to protect the eyesight of the many Ghanaians who are either unaware of their glaucoma status or not receiving care.

The background of the glaucoma patients suggests that most of them belong to formal religious groups. This information is useful as it provides a clue to bridging the unawareness gap by using these religious leaders as agents for educational campaigns. Studies have proven that opinion leaders remain a respected source of information necessary to influence decision making and behavioral change [20–22]. One major challenge is how to reach out to persons with glaucoma, to realize the much-needed early diagnosis critical to the prevention of vision loss associated with the disease.

There is reasonable support to integrate glaucoma screening and care into workplace health policies and protocols since most of the affected persons were in formal employment (Table 1). This strategy is necessary, as most people with glaucoma are unaware they have the disease [23–27]. The mean age of glaucoma cases was 47.30 years (range 9 to 86) and a loss of sight to glaucoma will have implications for productivity, quality of life, and family cohesion. Several studies have alluded to sex- and gender-based disparities in diseases in which eye diseases are no exception. There is renewed advocacy for sex-and gender-based studies to highlight the subtle disparities that are associated with a prevalent disease like glaucoma to inform a comprehensive planning and the objective prospect of case-finding approaches [28, 29]. Previous studies have reported the preponderance of glaucoma among males compared to their female counterparts [16]. In this study, there were more cases of glaucoma amongst males than females (Table 1) which if not well managed could have grave consequences for the livelihood of affected families. In most agrarian African communities, the traditional role of males is associated with breadwinning and any condition that affects a male’s role in providing the necessary financial support is worthy of attention [30]. This study, on the other hand, could have underrepresented women due to their generally low socioeconomic status which precludes them from accessing health care at the tertiary level [31, 32]. This suggests that efforts to stem sex-gender inequalities to access eye care should not be ignored.

Glaucoma pathogenesis has a genetic trace, nevertheless, most of the affected people are married [33, 34]. This implies that transmission from parent to children is inevitable. It is therefore necessary to consider screening for glaucoma as part of voluntary pre-marital marriage counseling for prospective couples as it is the case for sickle cell diseases [35–37]. This will ensure preventive genetic services for glaucoma control in Ghana. Also, periodic screening for first-generation progenies should be implemented for married people who have glaucoma. This will ensure early detection of glaucoma since controlled crosses may be difficult to achieve [38, 39].

The majority of the patients despite having essentially normal visual acuity were found to have lost significant peripheral vision as per the visual field assessment (Tables 3 and 4, Fig 1). The inherent threat is that given the most accessible and utilized means of transport in Ghana is by road, most of these patients who own cars per their socioeconomic status or engaged in commercial driving do not only endanger their own lives but that of other road users and passengers. Previous studies among commercial drivers in Ghana reported 14.5% of the history of road traffic accidents are due to poor judgment of distance [40]. Moreover, up to 7.7% of visual impairment among professional drivers is attributable to glaucoma [41]. This poor judgment stems mainly from restrictions of their visual fields. The spate of road accidents in Ghana is deemed as an emerging public health threat as it kills more people than most chronic and communicable diseases [42–45]. It is therefore imperative that a strict visual field assessment be incorporated into the pretest license requirement for the acquisition of drivers’ licenses in Ghana. The thinning of the retinal nerve fiber layer [46] as observed in this study is an indication that it is only a matter of time for those who currently have a glaucoma hemifield test that is “within normal limits” or “borderline” to lose essential peripheral vision.”

Contrary to an earlier assertion that Guans or people from the northern part of Ghana present with a severe form of glaucoma associated with large CDRs, there were not ethnic peculiarities with CDRs in this study [19]. Hence, screening among ethnic groups should be equitably distributed. Consistent with the literature, the cases of glaucoma were mainly of POAG (with its subtype, normal-tension glaucoma), bilateral, and adult-onset [16, 17]. These peculiarities of glaucoma among Ghanaians are a necessary guide for the screening, monitoring and tracking, and clinical characterization and management.

There is a gradual shift from the use of beta-blockers as the first line of treatment for glaucoma in Ghana as prostaglandins analogs are now listed as essential drugs in Ghana and are therefore covered by the National Health Insurance Scheme [18, 19]. That notwithstanding, there is still a comparable usage of beta-blockers (Table 5). The over-reliance on medical therapy, as opposed to surgical management, suggests poor knowledge of patients on treatment options, a lack of surgical glaucoma treatment options, and or lack of expertise in this domain. There is therefore the need to enhance education on treatment options and to update the skills of surgeons for the benefit of patients who may need such services.

## Conclusions

In summary, the epidemiological and clinical presentation of glaucoma at this tertiary care facility has a great lesson for public intervention. Counseling on consanguineous marriages, early referral, and mandatory eye screenings at the community level, work, and religious places can help identify people with glaucoma for prompt diagnosis and management.

## Supporting information

S1 Data (SAV)

## References

[1] Kingman S. Glaucoma is second leading cause of blindness globally. *Bull World Health Organ*. 2004: 82(11):811–890 PMC2623060 15640929

[2] Melamed C, Herndon L, Shaaraway T. The first African Glaucoma Summit vol.2010. August 6–7,2010, Accra, Ghana. http://www.worldglaucoma.org/AfricaSummit/index.php

[3] World Health Organization. Priority eye diseases.2019. www.who.int/blindess/causes/priority/en/index6.html

[4] Bowling B. *Kanski’s Clinical Ophthalmology*. 2016; 8th ed Elsevier limited: 319–333

[5] Faiq M, Sharma R, Dada R, Mohanty K, Saluja D, Dada T. Genetic, biochemical and clinical insights into primary congenital glaucoma. *J Curr Glaucoma Pract*. 2013; 7(2): 66–84 10.5005/jp-journals-10008-1140 26997785 PMC4741182

[6] He M, Foster PJ. Angle closure glaucoma in East Asian and European people. Different diseases? *Eye*. 2005; 20(1): 3–12 10.1038/sj.eye.6701797 15688051

[7] Kyari F, Abdull MM, Bastawrous A, Gilbert CE, Faal H. Epidemiology of glaucoma in sub-Saharan Africa: prevalence, incidence and risk factors. *Middle East Afr J Ophthalmol*. 2013; 20(2): 111–125. 10.4103/0974-9233.110605 23741130 PMC3669488

[8] Resnikoff S, Pascolini D, Etya’ale D, Kocur I, Pararajasegaram R, Pokharel GP, et al Global data on visual impairment in the year 2002. *Bull WHO*. 2004; 82: 844–851 doi: /S0042-96862004001100009 15640920 PMC2623053

[9] Quigley HA, Brohman AT. The number of people with Glaucoma worldwide in 2010 and 2020. *Br J Ophthalmol*. 2006; 90:262–267 10.1136/bjo.2005.081224 16488940 PMC1856963

[10] Sommer A, Tielsch JM, Katz J, Quigley HA, Gottsch JD, Javitt J, et al Relationship between intraocular pressure and primary open angle glaucoma among white and black Americans. The Baltimore eye survey. *Arch Ophthalmol*. 1991; 109:1990–1995 10.1001/archopht.1991.01080080050026 1867550

[11] Verrey JD, Foster A, Wormald R, Akuamoa C. Chronic glaucoma in northern Ghana- a retrospective study of 397 patients. *Eye (Lond)*. 1990; 4(1): 115–120 2323462 10.1038/eye.1990.14

[12] Herndon LW, Challa P, Ababio-Danso B, Boateng JO, Broomer B, Ridenhour P, et al Survey of glaucoma in an eye clinic in Ghana, West Africa. *J Glaucoma*. 2002; 11(5): 421–425. 10.1097/00061198-200210000-00009 12362082

[13] Ntim-Amponsah CT, Amoaku WMK, Ofosu-Amaah S, Ewusi RK, Idirisuriya-Khair R, Nyatepe-Coo E, et al Prevalence of glaucoma in an African population. *Eye (Lond)*. 2004; 18(5): 491–497 15131680 10.1038/sj.eye.6700674

[14] Guzek JP, Anyomi FK, Fiadoyor S, Nyonator F. Prevalence of blindness in people over 40 years in the Volta region of Ghana. *Ghana Med J*. 2005; 39(2): 55–62 10.4314/gmj.v39i2.35983 17299544 PMC1790811

[15] Otabil KB, Tenkorang SB, Mac AL, Otabil EA. Prevalence of Glaucoma in an eye clinic in Ghana. *Rus Open Med J*. 2013; 2(3): 0310.

[16] Budenz DL, Barton K, Tielsch JM. Prevalence of glaucoma in an urban West African population: the Tema eye survey. *JAMA Ophthalmol*. 2013; 131(5): 651–658. 10.1001/jamaophthalmol.2013.1686 23538512 PMC4139110

[17] Gyasi ME, Francis AW, Chen Y, Harrison RSR, Kodjo AR. Presentation of glaucoma in the Greater Accra metropolitan area of Ghana. *Ghana Med J*. 2014; 48 (3): 143–147. 10.4314/gmj.v48i3.4 25709123 PMC4335450

[18] Ocansey S, Kyei S, Diafo A, Darfor KN, Boadi-Kusi SB, Aglobitse PB. Cost of the medical management and prescription pattern for primary open angle glaucoma (POAG) in Ghana- a retrospective cross-sectional study from three referral facilities. *BMC Health Serv Res*. 2016; 16: 282 10.1186/s12913-016-1528-x 27430262 PMC4950601

[19] Nelson-Ayifah D, Mashige KP. Demographic and clinical characteristics of patients with glaucoma in a tertiary eye facility in Ghana. *Afr Vis Eye Health*. 2020; 79(1): a521.

[20] Carpenter CR, Sherbino J. How does an "opinion leader" influence my practice? *CJEM*. 2010; 12(5): 431–434. 10.1017/s1481803500012586 20880436 PMC3217217

[21] Flodgren G, Parmelli E, Doumit G, Gattellari M, O’Brien MA, Grimshaw J, et al Local opinion leaders: effects on professional practice and health care outcomes. *Cochrane Database Syst Rev*. 2011; 8: CD000125 10.1002/14651858.CD000125.pub4 21833939 PMC4172331

[22] Locock L, Dopson S, Chambers D, Gabbay J. Understanding the role of opinion leaders in improving clinical effectiveness. *Soc Sci Med*. 2001; 53(6): 745–757. 10.1016/s0277-9536(00)00387-7 11511050

[23] Tielsch JM, Sommer A, Katz J, Royall RM, Quigley HA, Javitt J. Racial variations in the prevalence of primary open-angle glaucoma. The Baltimore Eye Survey. *JAMA*. 1991; 266: 369–374. 2056646

[24] Mitchell P, Smith W, Attebo K, Healey PR. Prevalence of open-angle glaucoma in Australia. The Blue Mountains Eye Study. *Ophthalmology*. 1996; 103: 1661–1669. 10.1016/s0161-6420(96)30449-1 8874440

[25] Varma R, Ying-Lai M, Francis BA, Nguyen BB, Deneen J, Wilson MR, et al Prevalence of open-angle glaucoma and ocular hypertension in Latinos: the Los Angeles Latino Eye Study. *Ophthalmology*. 2004; 111: 1439–1448. 10.1016/j.ophtha.2004.01.025 15288969

[26] Mohammadi SF, Saeedi-Anari G, Alinia C, Ashrafi E, Daneshvar R, Sommer A. Is screening for glaucoma necessary? A policy guide and analysis. *J Ophthalmic Vis Res*. 2014; 9(1): 3–6. 24982725 PMC4074467

[27] Faal H. Primary open-angle glaucoma: everyone’s business. *Comm Eye Health*. 2012; 25(79): 41–43. 23520411 PMC3588135

[28] Clayton JA, Davis AF. Sex/gender disparities and women’s eye health. *Curr Eye Res*. 2015; 40(2): 102–9. 10.3109/02713683.2014.986333 25548854

[29] Khandekar R, Mohammed AJ. Gender inequality in vision loss and eye diseases: evidence from the Sultanate of Oman. *Indian J Ophthalmol*. 2009; 57(6): 443–449. 10.4103/0301-4738.57153 19861746 PMC2812763

[30] Poeze M. Beyond breadwinning: Ghanaian transnational fathering in the Netherlands. *J Ethnic Migrat Stud*. 2019; 45:16: 3065–3084.

[31] Neyhouser C, Quinn I, Hillgrove T, Chan R, Chhea C, Peou S, et al A qualitative study on gender barriers to eye care access in Cambodia. *BMC Ophthalmol*. 2018; 18(1): 217 10.1186/s12886-018-0890-3 30157788 PMC6116508

[32] Ofosu A, Osei I, Hagan M, Biekro L, Awedoba AK. Eye health knowledge and health-seeking behaviours in Ghana. *Afr Vision Eye Health*. 2018;77(1), a426.

[33] Wiggs JL, Pasquale LR. Genetics of glaucoma. *Hum Mol Genet*. 2017; 26(R1): R21–R27. 10.1093/hmg/ddx184 28505344 PMC6074793

[34] Wiggs JL. Genetic Etiologies of Glaucoma. *Arch Ophthalmol*. 2007; 125(1): 30–37. 10.1001/archopht.125.1.30 17210849

[35] Alhamdan NA, Almazrou YY, Alswaidi FM, Choudhry AJ. Premarital screening for thalassemia and sickle cell disease in Saudi Arabia. *Genet Med*. 2007; 9(6): 372–377. 10.1097/gim.0b013e318065a9e8 17575503

[36] Serjeant GR, Serjeant BE, Mason KP, Gibson F, Gardner R, Warren L, et al Voluntary premarital screening to prevent sickle cell disease in Jamaica: does it work? *J Community Genet*. 2017; 8(2): 133–139. 10.1007/s12687-017-0294-8 28251585 PMC5386916

[37] Alswaidi FM, O’Brien SJ. Premarital screening programmes for haemoglobinopathies, HIV and hepatitis viruses: review and factors affecting their success. *J Med Screen*. 2009; 16(1): 22–28. 10.1258/jms.2008.008029 19349527

[38] Griffiths AJF, Gelbart WM, Miller JH, Lewontin RC. *Modern Genetic Analysis* New York: Freeman W. H.; 1999 Human Pedigree Analysis. https://www.ncbi.nlm.nih.gov/books/NBK21257/

[39] Shalev SA. Characteristics of genetic diseases in consanguineous populations in the genomic era: Lessons from Arab communities in North Israel. *Clin Genet*. 2019; 95(1):3–9 10.1111/cge.13231 29427439

[40] Boadi-Kusi SB, Kyei S, Asare FA, Owusu-Ansah A, Awuah A, Darko-Takyi C. Visual function among commercial vehicle drivers in the central region of Ghana. *J Optom*. 2016; 9(1): 54–63. 10.1016/j.optom.2015.06.004 26364760 PMC4705311

[41] Ackuaku EM. Visual defects among professional drivers in Accra. *Ghana Med J*. 2000; 34(1): 24–31

[42] Gebru MK. Road traffic accidents: Human security perspective. Mekelle University, Ethiopia. *Int J Peace Dev Stud*. 2017; 8(2): 15–24.

[43] World Health Organization. *World Report on Road Traffic Injury Prevention*, Peden M. et al (eds.), World Health Organization, Geneva 2014

[44] World Health Organization *Global Status report on Road safety Time for Action* Switzerland; WHO Press, World Health Organization 2009 Geneva.

[45] World Health Organization *Pedestrian safety*: *A road safety manual for decision-makers and practitioners*. WHO Press, World Health Organization 2013.

[46] Ocansey S, Abu EK, Owusu-Ansah A, Mensah S, Oduro-Boateng J, Kojo RA, et al Normative values of retinal nerve fibre layer thickness and optic nerve head parameters and their association with visual function in an African population. *J Ophthalmol*. 2020; ID 7150673: 1–14. 10.1155/2020/7150673 PMC7036135 32104596

